# The role of oxidative stress in cardiovascular disease caused by social isolation and loneliness

**DOI:** 10.1016/j.redox.2020.101585

**Published:** 2020-07-16

**Authors:** Huige Li, Ning Xia

**Affiliations:** Department of Pharmacology, Johannes Gutenberg University Medical Center, 55131, Mainz, Germany

**Keywords:** Loneliness, Social isolation, Cardiovascular disease, Oxidative stress

## Abstract

Loneliness and social isolation are common sources of chronic stress in modern society. Epidemiological studies have demonstrated that loneliness and social isolation increase mortality risk as much as smoking or alcohol consumption and more than physical inactivity or obesity. Loneliness in human is associated with higher blood pressure whereas enhanced atherosclerosis is observed in animal models of social isolation. Loneliness and social isolation lead to activation of the hypothalamic-pituitary-adrenocortical (HPA) axis, enhanced sympathetic nerve activity, impaired parasympathetic function and a proinflammatory immune response. These mechanisms have been implicated in the development of cardiovascular disease conferred by social isolation although a causal relationship has not been established so far. There is evidence that oxidative stress is likely to be a key molecular mechanism linking chronic psychosocial stress to cardiovascular disease. NADPH oxidase-mediated oxidative stress in the hypothalamus has been shown to be required for social isolation-induced HPA axis activation in socially isolated rats. Oxidative stress in the rostral ventrolateral medulla is also a key regulator of sympathetic nerve activity. In the vasculature, oxidative stress increases vascular tone and promote atherogenesis through multiple mechanisms. Thus, preventing oxidative stress may represent a therapeutic strategy to reduce the detrimental effects of social stress on health.

## Highlights

• Loneliness and social isolation are common sources of chronic stress in modern society.
• Loneliness and social isolation increase the risk of cardiovascular disease.
• Oxidative stress is likely to be a key molecular mechanism linking chronic psychosocial stress to cardiovascular disease.
• NADPH oxidase-mediated oxidative stress is required for social isolation-induced HPA axis activation.
• In the vasculature, oxidative stress increases vascular tone and promote atherogenesis.

## Graphical abstract

**Figure:**
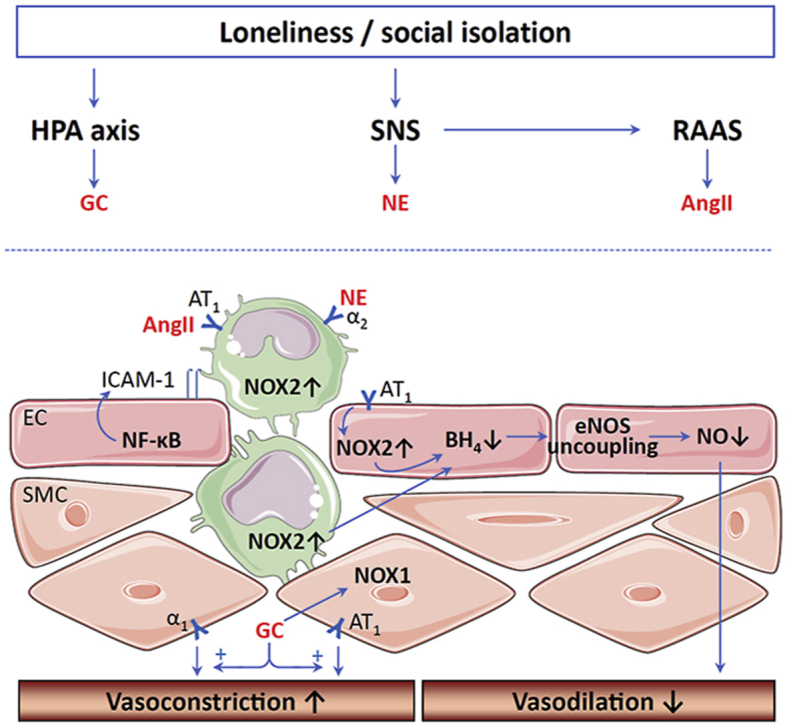

## 1 Introduction

Emerging evidence indicates that psychosocial stress is a strong independent risk factor for cardiovascular disease (CVD) [1]. The INTERHEART study has shown that psychosocial stress is the third most important modifiable risk factor for coronary heart disease and myocardial infarction, ranking only behind lipids and smoking [2,3]. Moreover, psychosocial stress accounts for approximately one-third of the attributable risk of coronary heart disease, making psychosocial stress is a strong cardiovascular risk factor [2,3].

Mental stress in modern human societies occurs largely during social interactions [4]. In addition to work-related stress [5], loneliness and social isolation represent major sources of chronic stress in humans [6,7]. Moreover, social and demographic changes in modern society have put an increasing number of individuals at risk for loneliness [[8], [9], [10], [11]].

## 2 Loneliness and social isolation cause cardiovascular disease in humans

There is clear evidence that strong social relationships increase the likelihood of survival [12] whereas loneliness and social isolation are associated with higher mortality [13,14]. The increased mortality risk is as high as light smoking or alcohol consumption and larger than that caused by obesity or hypertension [12,14]. Loneliness and social isolation are independent risk factors for CVD [15]. Lonely individuals have greater total peripheral vascular resistance [16,17] and higher risk to develop hypertension [18,19]. Consistently, loneliness and social isolation have been shown to increase the risks of coronary heart disease and stroke [20].

## 3 Social isolation promotes cardiovascular disease in animal experiments

Social isolation of experimental animals exacerbates atherogenesis. This has been shown in different atherosclerosis models. Individual housing of cynomolgus monkeys on an atherogenic diet increases atherosclerosis development in the coronary artery [21]. Likewise, increased atherosclerotic lesion has been found in socially isolated Watanabe heritable hyperlipidemic rabbits [22,23] and apolipoprotein E-knockout mice [24]. Interestingly, the enhanced atherosclerosis in animals exposed to social isolation is associated with physical inactivity, increased activity of the sympathetic nervous system (SNS), as well as enhanced inflammation and vascular oxidative stress [22,25,26].

## 4 Loneliness and social isolation lead to activation of the HPA axis

There is clear evidence that loneliness leads to activation of the HPA axis in humans [27,28]. The HPA axis is the main producer of glucocorticoids, including cortisol in humans and corticosterone in rodents. The cortisol production follows the circadian rhythm with higher levels in the morning and lower levels in the evening. Lonely individuals have greater morning cortisol increases [29], elevated circulating cortisol concentrations [30,31], and impaired glucocorticoid receptor (GR) sensitivity [32,33], suggesting that loneliness causes overaction of the HPA axis [34].

Activation of the HPA axis has also been observed in animal models of social isolation. Monogamous prairie voles build pair bonds with mating partners and are used as an animal model to study the consequences of pair bonding or partner loss [35]. Separation of pair-bonded prairie voles from the partner leads to an elevation of circulating corticosterone concentrations [[35], [36], [37]]. In contrast, separation from a same-sex sibling has no effect on corticosterone levels [35]. The separation of pair-bonded prairie voles is associated with an increase of corticotrophin-releasing hormone and adrenocorticotropic hormone [[35], [36], [37]], indicating an activation of the entire HPA axis.

In the vascular system, glucocorticoids can promote the development of hypertension and atherosclerosis by augmenting vasoconstriction, reducing endothelial nitric oxide (NO) production and enhancing oxidative stress. Glucocorticoids potentiate the effects of catecholamines and other vasoconstrictors on vascular smooth muscle cells [38,39]. In endothelial cells, glucocorticoids decrease NO production by down-regulating the expression of endothelial NO synthase (eNOS) [40]. Moreover, glucocorticoids also reduce eNOS enzymatic activity by reducing eNOS phosphorylation at serine 1177 [41]. Conversely, siRNA-mediated knockdown of glucocorticoid receptor increases eNOS expression and NO production in endothelial cells [42]. Mice deficient in eNOS do not develop hypertension in response to glucocorticoids, supporting the crucial role of reduced endothelial NO production in the development of glucocorticoid-induced hypertension [40,43,44]. In socially isolated prairie voles, the endothelium-dependent vasodilation is decreased [45], indicating a reduced endothelial NO production caused by social isolation, although the role of glucocorticoids in this effect is still unknown.

In addition to its role in blood pressure regulation, endothelial NO also represents a key anti-atherosclerotic factor [[46], [47], [48]]. Thus, the reduced endothelial NO production caused by glucocorticoid is likely also involved in the atherogenic effects of social isolation.

## 5 Dysregulation of the autonomic nervous system

The findings on SNS activation in loneliness and social isolation is less consistent than the activation of the HPA axis [27,28]. Chronic social isolation in humans and macaques is associated with increased urinary levels of norepinephrine metabolites but not epinephrine [31] (Table 1). The effect of social isolation on local catecholamine concentrations in SNS-innervated tissues seems to be greater than that on systemic catecholamine levels. In ovarian cancer patients, poor social support is associated with higher norepinephrine levels in tumor tissues as compared to patients who have strong social networks. However, no difference in plasma norepinephrine has been found in the same patient population regarding to social support [49,50]. The local norepinephrine in the tumor tissue is implicated in local inflammation, metastasis and tumor cell proliferation mediated by β-receptors [[49], [50], [51], [52]].

**Table 1:** Dysregulation of the autonomic nervous system.

Species	Model	Effects	References
Human	Perceived social isolation	Elevated urinary levels of norepinephrine metabolites	[31]
Macaques	Perceived social isolation	Elevated urinary levels of norepinephrine metabolites	[31]
Ovarian cancer patients	Poor social support	Higher norepinephrine levels in tumor tissues	[49,50]
Rats	Chronic social isolation	Increased plasma epinephrine & norepinephrine	[53]
Prairie voles	Social isolation	Increased sympathetic and decreased parasympathetic drive to the heart	[36]
Prairie voles	Social isolation	Lower HF-HRV	[67,68]
Human	Lower social integration	Lower HF-HRV	[59,66]

In adult rats, chronic social isolation has been found to increase the plasma concentrations of both epinephrine and norepinephrine [53]. Social isolation of male prairie voles from the bonded partner results in increased heart rate, heart rhythm dysregulation, and autonomic imbalance characterized by increased sympathetic and decreased parasympathetic drive to the heart [36].

A well balanced vagal system is important to prevent the detrimental effects of a SNS overactivation [54,55]. The vagally-mediated parasympathetic activity can be monitored with the high frequency heart rate variability (HF-HRV), which denotes heart rate variations associated with respiration [56]. The vagal outflow is inhibited by the cardiovascular center during inhalation and restored during exhalation [56]. Although the heart is innervated both by the SNS and the vagal system, the effect of the SNS is too slow to modify the beat-to-beat changes [57]. As a result, the HF-HRV can be considered a direct measure of the parasympathetic control of the heart [58,59]. A lower HF-HRV is correlated with depression, reduced cognitive function, CVD, and all-cause mortality [[60], [61], [62], [63]].

It has been reported that social environment has an impact on HF-HRV [59]. High HF-HRV is observed in married individuals [64], and even higher HF-HRV in people with happy marriage life [65]. In the Whitehall cohort in UK, a smaller HF-HRV has been found to be associated with a lower social integration [66] (Table 1). A similar link between social integration and the autonomic nervous system has been shown for students moving to other countries to study. The poor social integration in the initial time period in a foreign county is associated with higher heart rate and lower HF-HRV [59].

Regulation of HF-HRV by social environment has also been shown in animal studies. Compared to socially paired prairie voles, voles suffer from social isolation have lower HF-HRV, both at baseline and in response to stress [67,68]. The poor HF-HRV in socially isolated voles can be normalized by a treatment with exogenous oxytocin [68]. Interestingly, loneliness and social isolation can change the response to oxytocin treatment. In young healthy adults, oxytocin increases both sympathetic and autonomic cardiac control [58]. In lonely persons, however, the effects of oxytocin on HF-HRV are reduced, leading to a shift towards relative SNS overaction [58]. Thus, the dysregulation of the autonomic nervous system may represent one of the pathomechanisms underlying the detrimental effects of social isolation on health, because the parasympathetic nervous system activity is required to counterbalance the sympathetic output and its deleterious effects [[57], [58], [59]].

## 6 Proinflammatory response of the immune system

Loneliness and social isolation in humans cause differentiated gene expression in circulating leukocytes leading to the so-called conserved transcriptional response to adversity (CTRA). The CTRA is characterized by up-regulation of proinflammatory genes and down-regulation of antiviral immunity-related genes resulting in enhanced inflammation and an impaired antiviral response [32,69,70]. Loneliness leads to a selective expansion of the immature classical monocyte subset without changing the number of total circulating leukocytes [31,71]. The increased circulating monocyte frequencies and percentages are the main source of the proinflammatory function of the CTRA [69]. The CTRA has been observed in both humans and macaques with high perceived social isolation [31,32,71].

The proinflammatory CTRA observed in individuals exposed to loneliness and social isolation is likely to be caused by the SNS activation rather than the HPA axis [32,69]. Loneliness increases the levels of norepinephrine metabolites (but not epinephrine) in urine [31]. In mouse models of repeated social defeat [69] and chronic variable stress [72], SNS activation has been shown to enhance myelopoiesis leading to an increased output of neutrophils and inflammatory monocytes. The effects can be prevented by pharmacological antagonism or genetic disruption of the β3-adrenoreceptor [69,72], indicating a causal role of these receptors. However, this mechanism (SNS-mediated myelopoiesis and CTRA) has not been verified in the mouse model of social isolation, so far.

Remarkably, the CTRA-induced proinflammatory monocytes can traffic into the brain, cause “sickness behaviors” and augment loneliness, creating a vicious cycle [31,73]. The CTRA and proinflammatory response are likely to represent crucial mechanisms mediating the harmful health effects. Recent epidemiologic studies have associated CTRA with increased risk of cardiovascular, metabolic, and neoplastic diseases [74,75].

Moreover, chronic psychosocial stress has been associated with elevated levels of circulating proinflammatory cytokines, particularly of IL-6. Socially less well connected individuals have higher IL-6 levels [76,77].

## 7 Oxidative stress in the hypothalamus is required for HPA activation

Oxidative stress is a key molecular mechanism linking chronic psychosocial stress to cardiovascular disease [78]. In socially isolated animals, oxidative stress has been observed both in the brain and in peripheral tissues. In the brain, oxidative stress is required for the social isolation-induced HPA activation. In the vascular tissue, oxidative stress is likely to be the result of social isolation-induced activation of HPA, SNS and proinflammatory immune response. In supporting this concept, a recent clinical study has demonstrated a dose-dependent association between of HPA activation, SNS activation and inflammation and oxidative damage [79].

In the rat model of social isolation rearing, the animals are housed individually starting at the age of 21 days. The protein level of corticotropin-releasing factor in the hypothalamus and the concentration of adrenocorticotropic hormone in the plasma are increased after 4 weeks of isolation, whereas the increased corticosterone levels in plasma and saliva can be observed after 7 weeks [80]. In contrast, oxidative stress markers in the hypothalamus are increased as early as two weeks after social isolation [80]. The enhanced oxidative stress in the brain of socially isolated rats is attributable to an induction of NADPH oxidases (NOX) [80,81].

NOX are a family of reactive oxygen species (ROS)-generating enzymes [47]. The enzyme complex consists of two membrane-associated (a NOX protein and p22phox) and several cytoplasmic (p40phox, p47phox, p67phox, and rac1) subunits. Among the NOX isoforms expression in the brain, a clear up-regulation of NOX2 is seen in the hypothalamus and prefrontal cortex of socially isolated rats, whereas no changes in the expression of NOX1, NOX3 and NOX4 have been found [80,81]. The activity of NOX2 requires translocation of the cytoplasmic regulatory subunits to the membrane which is initiated by p47phox phosphorylation [82,83]. Interestingly, the expression of NOX2 components, such as p22phox, p67phox, p47phox, and p40phox, is increased by social isolation as well [81]. Moreover, the induction of NOX2 expression is an early event (two weeks after isolation) that precedes the activation of the HPA axis [80]. Treatment with the NOX inhibitor apocynin prevents social isolation-induced PHA activation [80,81]. In addition, rats with a loss-of-function mutation in p47phox, which is an essential component of the NOX2 complex, are protected from social isolation-induced oxidative stress, HPA activation and behavior changes [80]. These results indicate that NOX2-mediated oxidative stress is an early trigger of HPA activation and is causally involved in social isolation-induced pathology.

**Fig. 1:**
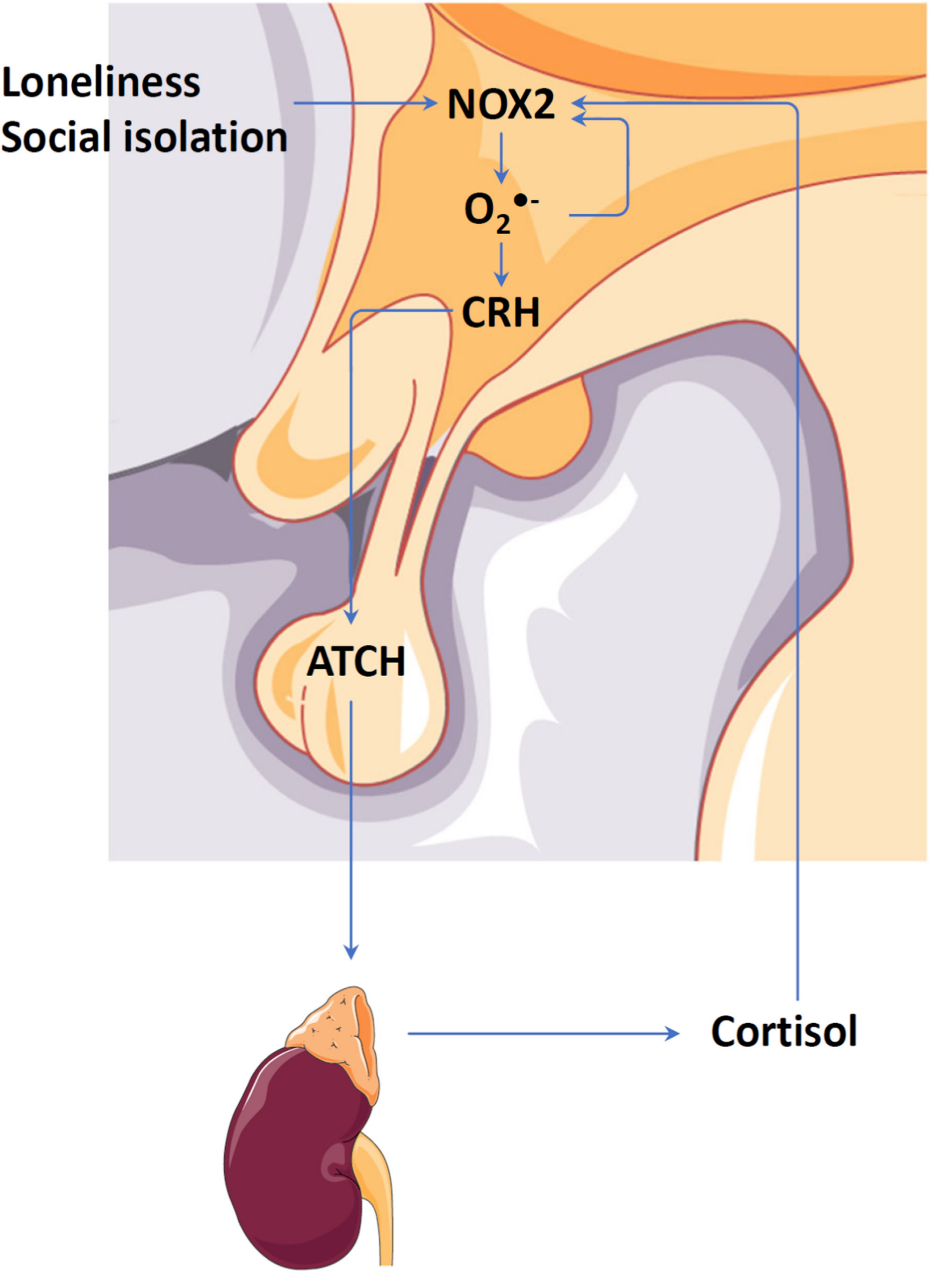
**Role of oxidative stress in social isolation-induced HPA activation.** Social isolation increases the expression of NOX2 and its components in the hypothalamus before the elevation of corticotropin-releasing hormone (CRH) in the hypothalamus and the concentration of adrenocorticotropic hormone (ACTH) in the plasma. Inhibition of NOX2 activity prevent the activation of the hypothalamic-pituitary-adrenocortical (HPA) axis and the NOX2 up-regulation, indicating a positive feedback mechanism. NOX2 expression in the hypothalamus may be further potentiated by glucocorticoids produced by the adrenal gland. The illustrations of anatomical structures were retrieved from Servier Medical Art licensed under the Creative Commons Attribution 3.0 Unported License and have thereafter been assembled and processed.

Interestingly, the NOX2 induction by social isolation in the hypothalamus is prevented by apocynin treatment and absent in rats with p47phox mutation [80], suggesting a positive feedback mechanism of social isolation-induced oxidative stress in the brain (Fig. 1).

A second positive feedback mechanism may be the HPA axis itself. Although glucocorticoids have been shown to inhibit NOX-mediated ROS production in some cell types [84], dexamethasone has been shown to up-regulate NOX expression in hippocampal neurons [85]. Moreover, the dexamethasone-induced ROS production in hippocampal neurons can be prevented by GC receptor blockade, indicating a specific effect of glucocorticoids. Nevertheless, this experiment was performed using organotypic hippocampal slice cultures [85]. It is still unclear whether it applies to in vivo situations in social isolation animals.

In addition, a down-regulation of antioxidant enzymes may also contribute to the brain oxidative stress induced by social isolation [84,86] although the results in this regard are less consistent [11] (Table 2).

**Table 2:** Changes of antioxidant enzymes.

Species	Age at isolation	Duration of isolation (weeks)	Changes	References
Rats	3 weeks	7	↓: Prdx1, Ucp-1 (in visceral fat)	[122]
			↑: Nox1, Hmox-1, Adrb3 (in visceral fat)	
Rats	3 weeks	8	↓: catalase, peroxidase (GPx) and SOD (in the rat hippocampus)	[111]
Rats	2–3 months	3	↓: SOD1 nuclear fraction (in cerebral cortex)	[123]
			↔: SOD1 (in hippocampus)	
Rats	2.5 months	3	↓: GSH (in hippocampus)	[124]
			↔: MDA; SOD activity (in hippocampus)	
Rats	3 months	3	↑: SOD, catalase (in hippocampus)	[125]
Rats	3 months	3	↓: GPx activity (in hippocampus)	[126]
			↔: GPx expression; SOD, catalase, GLR activity (in hippocampus)	
			↑: GLR expression (in hippocampus)	
Rats	3 months	3	↔: GLR, GSTA3 (in hippocampus)	[127]
			↑: GCLM (in hippocampus)	

## 8 Oxidative stress in the brain enhances SNS activity

ROS stimulates central and peripheral SNS activity [87]. The sympathetic nervous control center is located in the rostral ventrolateral medulla (RVLM). Redox status in the RVLM is crucial in regulating the sympathetic outflows and blood pressure [88]. Induction of oxidative stress in the RVLM increases sympathetic outflow and elevates blood pressure [89,90]. On the contrary, reducing ROS levels in the RVLM inhibits SNS activity and lowers blood pressure [90].

The sympathoexcitation induced by oxidative stress in the RVLM has been shown to play a crucial role in causing blood pressure elevation in a number of hypertension models, including obesity-induced hypertension [91], neurogenic hypertension [92], angiotensin II (AngII)-induced hypertension [93] as well as in the spontaneously hypertensive rats [94].

NADPH oxidases seem to play a key role in mediating RVLM oxidative stress. Bilateral microinjection of AngII into the RVLM leads to oxidative stress and blood pressure elevation [93]. These effects are mediated by the angiotensin receptor subtype 1 (AT1) and can be prevented by inhibiting NADPH oxidase [93]. The main hypertension-related RVLM isoform is NOX2 [95]. Neurogenic hypertension in phenol-injected rats is associated with up-regulation of NOX2 and its components in the medulla [92]. It has been shown recently that acupuncture reduces SNS activity and lowers blood pressure in the spontaneously hypertensive rats [90]. Among the NOX isoforms expressed in RVLM, the beneficial effects of acupuncture are associated with a down-regulation of NOX2, but not NOX1 or NOX4. The sympatholytic and antihypertensive effects of acupuncture can be mimicked by RVLM administration of NOX inhibitors. Moreover, activation of RVLM NOX abolishes protective effects of acupuncture supporting the conclusion that the antihypertensive effects of acupuncture are mediated by buffering NOX2-mediated oxidative stress [90].

**Fig. 2:**
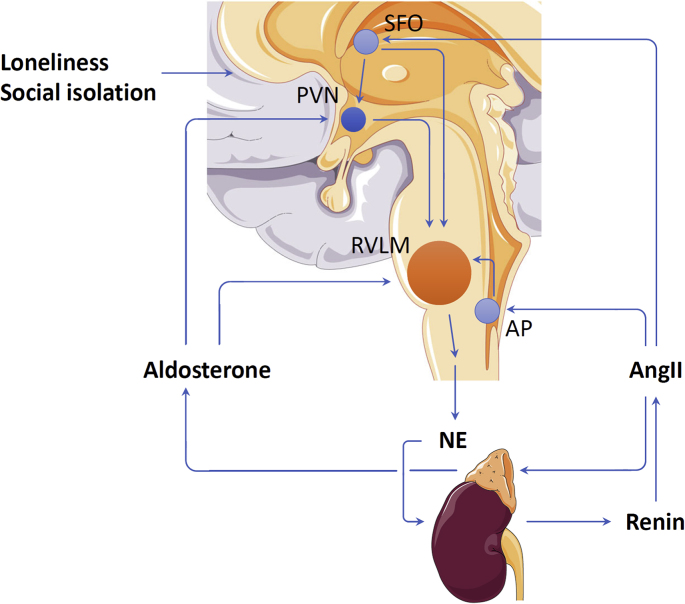
**Role of oxidative stress in regulating sympathetic nerve activity.** NOX2-mediated oxidative stress in the rostral ventrolateral medulla (RVLM) is crucial in regulating the sympathetic outflows and blood pressure. This has been shown in numerous hypertension models but not in models of social isolation so far. Social isolation leads to up-regulation of NOX2 gene expression in several brain regions; it is yet still unknown whether this also occurs in RVLM. Theoretically, SNS stimulation leads to activation of the renin-angiotensin system resulting elevated levels of circulating angiotensin II (AngII) and aldosterone. Aldosterone can penetrate blood–brain barrier and induce NOX2 expression in the paraventricular nucleus (PVN), perhaps also in RVLM. AngII, on the other hand, can cause oxidative stress in blood-brain barrier-lacking circumventricular organs, such as area postrema (AP) and subfornical organ (SFO). Oxidative stress in such structures may spread to the RVLM via direct diffusion of oxidants or indirect mechanisms. SFO oxidative stress can also cause PVN activation. PVN neurons, in turn, enhance SNS activity by stimulating sympathetic preganglionic neurons in the spinal cell column and by activating cells in the RVLM. The illustrations of anatomical structures were retrieved from Servier Medical Art licensed under the Creative Commons Attribution 3.0 Unported License and have thereafter been assembled and processed.

Although the role of RVLM oxidative stress in regulating SNS activity is clearly shown, it is yet still unknown whether this mechanism is involved in social isolation-induced SNS activation. In the rat model of social isolation rearing, increased NOX2 gene expression has been shown in specific brain areas: amygdala, hippocampus, nucleus accumbens and prefrontal cortex, but not striatum [81]. NOX2 expression was not analyzed in RVLM in that study and needs to be addressed in future investigations. Nevertheless, RVLM oxidative stress under the condition of social isolation is conceivable. SNS activation caused by social isolation may leads to activation of the renin-angiotensin system (RAS) resulting elevated levels of circulating AngII and aldosterone. Although AngII cannot penetrate the blood-brain-barrier, circulating AngII can stimulate AT1 receptor and cause oxidative stress in blood–brain barrier-lacking circumventricular organs, such as Area postrema (AP), organum vasculosum laminae terminalis (OVLT) and subfornical organ (SFO) [95,96]. Theoretically, oxidative stress may spread from these structures to the RVLM via direct diffusion of oxidants or indirect mechanisms [95]. Moreover, neurons in the SFO send axonal projections to the paraventricular nucleus (PVN) of the hypothalamus (Fig. 2). PVN neurons, in turn, enhance SNS activity by stimulating sympathetic preganglionic neurons in the spinal cell column and by cells in the RVLM [96]. In addition, a complete endogenous RAS with the all components is expressed in the brain including PVN. Unlike AngII, plasma aldosterone can penetrate blood-brain-barrier and reach PVN leading to up-regulation of ACE and AT1 expression, enhanced superoxide production mediated NADPH oxidases and sympathetic hyperactivation [97]. Furthermore, the RVLM itself has high density of ACE and AT1 [96]. AngII and other proinflammatory stimuli enhance the expression of ACE, AT1 receptor, but also NOX2 resulting in oxidative stress and higher SNS activity [98]. Nevertheless, these mechanisms remain to be shown in models of social isolation.

## 9 Oxidative stress in the vasculature

**Fig. 3:**
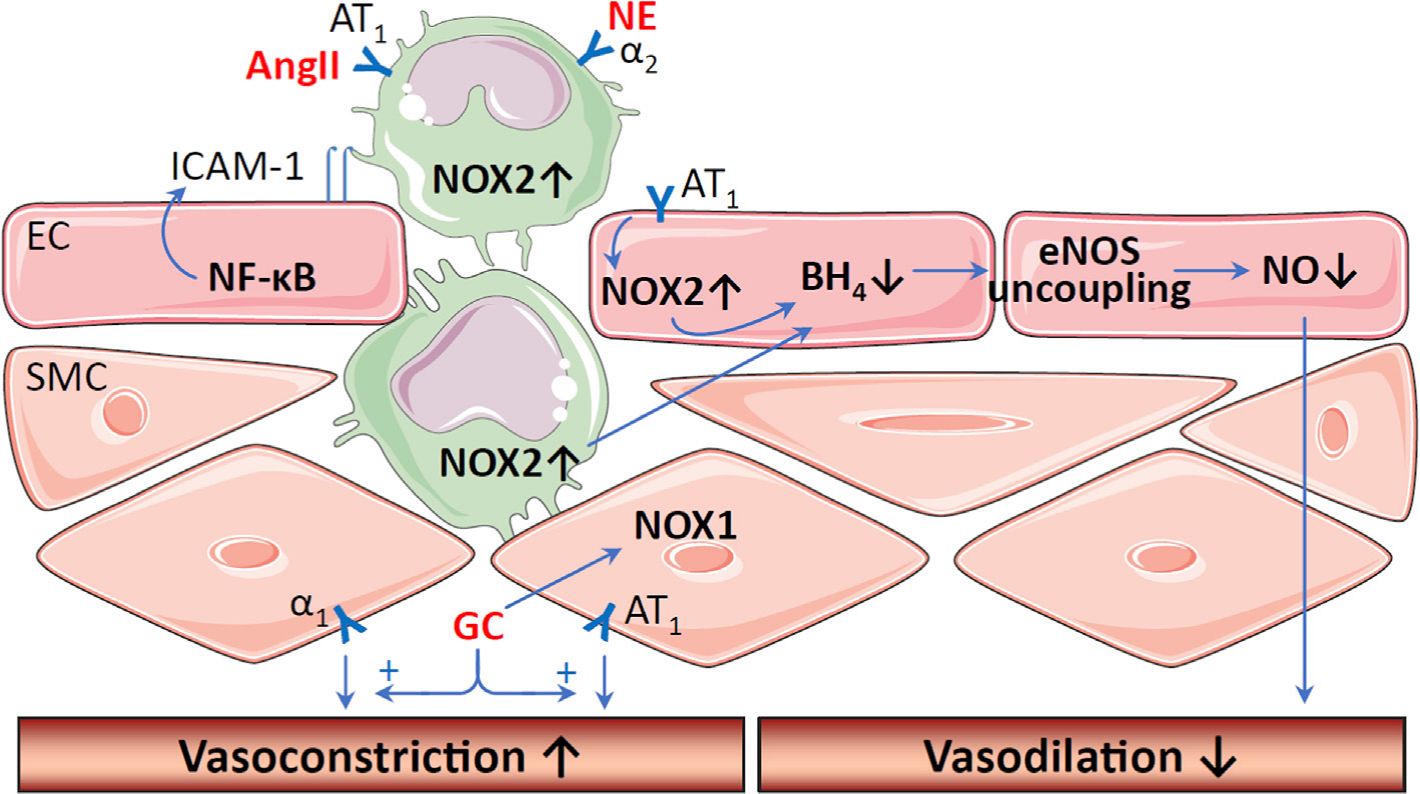
**Social isolation-induced oxidative stress in the vasculature.** Social isolation leads to activation of the HPA axis and the SNS. SNS stimulates renin secretion and the production of angiotensin II (AngII). AngII activates NOX2 in endothelial cells (EC) causing oxidative stress, which may induce uncoupling of the endothelial nitric oxide synthase (eNOS) through tetrahydrobiopterin (BH_4_) oxidation. Oxidative stress in EC also leads to activation of NF-κB and the induction of adhesion molecules resulting in vascular inflammation. AngII and the SNS neurotransmitter norepinephrine (NE) stimulate NOX2-medaited superoxide production via AT_1_ and α_2_ receptors, respectively, and promote the infiltration of immune cell into the vascular wall. Glucocorticoids (GC) enhance NOX1 expression in vascular smooth muscle cells (SMC) and potentiate vasoconstriction induced by NE (via α_1_ receptor) or AngII (AT_1_ receptors). Moreover, GC also reduce eNOS gene expression and serine 1177 phosphorylation resulting in decreased NO production and impaired vasodilation. The scheme is partly adopted from our previous publications ([11,120]). The images of monocytes, EC and SMC used in this figure are from Servier Medical Art licensed under the Creative Commons Attribution 3.0 Unported License [121].

Social isolation leads to oxidative stress in the vascular tissue (Fig. 3). Higher NADPH oxidase activity is observed in the aortic arch of individually caged Watanabe heritable hyperlipidemic rabbits [25]. NADPH oxidases represent the major ROS producers in blood vessels and the NADPH oxidase-mediated oxidative stress promotes atherosclerosis [47,82,83]. Indeed, increased atherosclerotic lesions have been found in socially isolated Watanabe heritable hyperlipidemic rabbits [22] as well as apolipoprotein E-knockout mice [24]. In cell culture experiments, treatment with oxytocin reduces NADPH oxidase activity in endothelial cells, smooth muscle cells, monocytes and macrophages and endothelial cells [99]. Oxytocin also inhibits proinflammatory cytokine secretion from endothelial cells and macrophages [99]. Chronic in vivo administration with oxytocin attenuates atherosclerosis lesion in socially isolated animals (Table 3), both in Watanabe heritable hyperlipidemic rabbits [100] and in socially isolated apolipoprotein E-knockout mice [26]. Although it is not clear to what extent the anti-atherosclerotic effect of oxytocin is attributable to the inhibition of NADPH oxidase activity, it is conceivable that the reduction of oxidative stress contributes to the vasoprotective effect of the molecule.

**Table 3:** Experimental therapies.

Species	Model	Treatment	Effects	Ref.
Rats	Social isolation rearing	clozapine or N-acetyl cysteine	Improvement of mitochondrial, immunological, neurochemical, and behavioral deficits	[110]
Mice	Postweaning social isolation	apocynin	Partial alleviation of schizophrenia-like behavioral phenotypes	[128]
Rats	Chronic social isolation	olanzapine	Protection from hepatic protein oxidation and improvement of antioxidant defense	[129]
prairie vole	Chronic social isolation	oxytocin	Prevention of glucocorticoid increase, oxidative damage, telomere degradation and anhedonia	[130]
Mice	Chronic social isolation	oxytocin	Reduced atherosclerosis; less IL-6 from adipose tissue; no changes in plasma lipids	[26]
Rabbits	Chronic social isolation	oxytocin	Reduced atherosclerosis & CRP; no changes in plasma lipids	[100]

CRP, C-reactive protein.

Mechanistically, social isolation-induced vascular oxidative stress can be mediated by HPA axis, SNS activation, and inflammatory cells (Fig. 3). The effects of glucocorticoids on NADPH oxidase are likely to be cell type- and context-dependent [84,101]. Treatment with dexamethasone has been shown to enhance the expression of NOX1 in cultured vascular smooth muscle cells as well as in vascular tissues in vivo [102].

SNS activation may also contribute to social isolation-induced vascular oxidative stress. Enhanced systemic oxidative stress has been observed in rats treated in vivo with the sympathetic neurotransmitter norepinephrine [103]. Treatment of isolated human peripheral blood mononuclear cells (PBMCs) with norepinephrine leads to up-regulation of NOX2 and its component proteins p22phox and p67phox resulting in enhanced superoxide production [104]. The norepinephrine-stimulated superoxide production is mainly mediated by the α2-receptor and involves protein kinase C activity [104]. In addition, treatment of monocytes with norepinephrine enhances their adhesion capacity with endothelial cells, indicating a proinflammatory phenotype [104]. This is consistent with the finding that SNS activation in lonely individuals elevates the number of proinflammatory monocytes [31]. In agreement, a recent study has shown that proinflammatory monocytes infiltrate into the blood vessels and induce local inflammation and oxidative stress, mechanisms that are crucial for the development of cardiovascular disease [105]. In this sense, the pathways triggered by social isolation resemble those stimulated by other environmental stressors, such as traffic noise, emotional stress or air pollution [[106], [107], [108], [109]]. The converging common mechanism leading to cardiovascular disease is an induction of vascular inflammation and oxidative stress [109].

## 10 Mitochondrial ROS

In addition to NADPH oxidase, increased ROS production by mitochondria also contributes to social isolation-induced oxidative stress [84]. The mitochondrial respiratory chain is a major source of cellular ROS that is counterbalanced by glutathione and endogenous antioxidant systems [110]. Deficiency of the mitochondrial antioxidant capacity results in disruption of ATP synthesis and oxidative damage [110]. Chronic social isolation inhibits the activities of antioxidant enzymes catalase, glutathione peroxidase and superoxide dismutase in the rat hippocampus [111,112]. Juvenile social isolation stress induces mitochondrial dysfunction in adulthood, evidenced as impaired respiratory chain complex resulting in mitochondrial ROS formation, oxidative damage and ATP reduction in both brain and heart [113]. Chronic social isolation in rats inhibits mitochondrial oxidative metabolism by ROS-dependent inhibition of citric acid cycle enzymes containing redox-sensitive active sites [114]. Mitochondrial dysfunction and oxidative stress have also been observed in hippocampus of mice subjected to early social isolation stress [115]. In female rhesus macaques, social interactions have been shown to influence mitochondrial DNA copy number in immune cells [116].

The exact mechanisms how mitochondrial ROS participate in social isolation-induced pathology are incompletely understood. It has been shown that mitochondrial ROS promote the production of proinflammatory cytokines such as IL-6 and TNF-α [117]. Moreover, there exists a cross-talk between mitochondrial ROS and NADPH oxidase-derived ROS, resulting in an amplification mechanism for cellular oxidative stress [118,119]. The involvement of such mechanisms in social isolation-induced cardiovascular disease, however, remains elusive.

## 11 Summary and future directions

There is clear evidence that loneliness and social isolation impair cardiovascular health. The underlying mechanisms may include HPA activation, SNS hyperactivity, parasympathetic dysfunction and a proinflammatory immune response. Oxidative stress is the brain is required for the social isolation-induced HPA activation and is probably also involved in SNS activation. In the vasculature, oxidative stress impairs endothelial function and promotes atherosclerosis. Both in the central nervous system and in the peripheral, NOX2 seems to be a key superoxide producer mediating social isolation-induced oxidative stress. This, however, remains to be validated in future studies using transgenic animal models. Another point is that some mechanisms presented in the present review article are puzzled together from studies using different models. Whether these mechanisms apply to social isolation remains elusive.

## Declaration of competing interest

The authors declare that they have no conflicts of interest.
